# The Use of Genomic Information for the Conservation of Animal Genetic Diversity

**DOI:** 10.3390/ani11113208

**Published:** 2021-11-10

**Authors:** J. Kor Oldenbroek

**Affiliations:** Centre for Genetic Resources, the Netherlands, Wageningen University & Research, P.O. Box 338, 6700 AH Wageningen, The Netherlands; kor.oldenbroek@wur.nl

**Keywords:** genetic diversity, conservation, genomics

## Abstract

The conservation of genetic diversity, both among and within breeds, is a costly process. Therefore, choices between breeds and animals within breeds are unavoidable, either for conservation in vitro (gene banks) or in vivo (maintaining small populations alive). Nowadays, genomic information on breeds and individual animals is the standard for the choices to be made in conservation. Genomics may accurately measure the genetic distances among breeds and the relationships among animals within breeds. Homozygosity at loci and at parts of chromosomes is used to measure inbreeding. In addition, genomics can be used to detect potentially valuable rare alleles and haplotypes, their carriers in these breeds and can facilitate in vivo or in vitro conservations of these genomic regions.

Genetic diversity is the set of differences between species, breeds within species, and individuals within breeds present in their DNA or observed in animals as a consequence [1]. Genetic diversity between breeds depends of the number of breeds and the size of their populations. Genetic diversity within a breed is influenced by genetic drift, selection, migration, and mutation. Genetic diversity is not static. Between breeds, it is threatened by the upgrade and replacement of local breeds by highly selective main stream breeds [2]. Within breeds, it is threatened by an intense selection for a few traits and an increase in the genetic relationship among the animals that results in inbreeding and its negative aspects. The maintenance of genetic diversity between breeds is important because breeds differ in adaptive traits with respect to surviving and reproducing in specific environments and to responding to different market requirements [3]. Moreover, you need different breeds to apply crossbreeding that may increase the efficiency of a production system. Within breeds, the maintenance of genetic diversity is important because it determines to a great extent the possibilities for selection, and it avoids inbreeding [2]. The threats for genetic diversity between breeds resulted in a global situation in the past 50 years where a lot of local breeds are at risk [4]. In the ideal world, a conservation program would then be developed for each of the breeds at risk. For most countries, however, the costs required for conserving all breeds at risk will be greater than the resources available for conservation. Therefore, a decision has to be taken with respect to which breeds will be utilized and which breeds should be conserved. The genetic diversity within a breed and its genetic relationship with other breeds might be one of the decisive criteria for conservation [4]. 

In the past, these criteria could only be based on the history of breeds and pedigree analysis. The starting-point for these pedigree-based calculations is that each individual receives a random sample of 50 percent of the alleles of its parents in the random process of meiosis. Nowadays, in the era of genomics, DNA-analysis of individuals and parents provides full insight into which alleles of an individual came from its sire and which came from its dam. The measures of genetic relationships and inbreeding based on pedigrees are expectations, while molecular genetic estimates of inbreeding are the particular realisations of such expectations [5]. Therefore, molecular genetic-based measures of genetic relationships between breeds measure more accurately the genetic distances among breeds. The costs of generating genomic data on a large scale are becoming increasingly affordable. For these reasons, genomic information became the standard for the choices to be made in the conservation of farm animal genetic diversity [6].

Inbreeding can be more accurately established with genomics than with pedigree-based measures. Runs of Homozygosity (ROH) play an important role in these measures. Runs of homozygosity are contiguous lengths of homozygous genotypes that are present in an individual due to parents transmitting identical haplotypes to their offspring. Haplotypes are contiguous loci in a very high linkage equilibrium. The length of these ROH may reflect either the diversity in the population or the mating practices, or both. Long ROHs are the result of recent inbreeding, while short ROHs are signs of inbreeding in the past. The accuracy of molecular measures is also higher because pedigree errors are easily detected. Genomics can also be used to detect potentially valuable rare alleles and haplotypes and their carriers in these breeds and promote the conservation of these genomic regions. Nowadays, genomic management is an important tool in breeding programs for livestock animals in large and in small populations [7].

In the conservation of genetic diversity, a lot of attention is given to the conservation of breeds within species. In the genomics era, Sponenberg [8] defined a breed as a population of animals with a repeatable genomic package and a reasonably high level of predictability of performance. Thus, a breed is a unique combination of alleles and haplotypes created by many generations of natural and/or artificial selection. The conservation of breeds deserves attention because breed variation roughly accounts for approximately half the total genetic variation within a species [9]. 

The conservation of a breed can be realized in vivo: by keeping animals of the breed alive. Numerically, small breeds can still fulfil a sustainable role in society in the utilisation of rural areas [3]. However, for the conservation of live in vivo populations, it is important that the small breed remains an attractive alternative to many other breeds, which are continuously evolving. It is important that a small breed also continues to evolve and improves genetically over time [7]. Genomic data are an important tool for their genetic improvement. Small breeds benefit from the genomic infrastructure present for other cattle populations. It is expected that these genomic tools are more effective in improving a small breed and in managing inbreeding simultaneously compared to the traditional tools. Although many local livestock breeds are “at risk” on the basis of the number of purebred breeding females in a breed registry, there is often a reservoir of unregistered animals that may belong to the same breed. Genetic tests can unequivocally determine the breed origin of cattle without pedigree data [10]. Such a test provides the possibility to incorporate animals without pedigree data in the population of purebred animals.

The conservation of genetic diversity can also be realized by building cryo-collections of germ plasm. In the past 50 years, in many countries, farm animal gene banks are established that mainly conserved cryo-conserved semen. Gene bank collections might be used to re-establish an extinct breed. Moreover, it can be used to support the live conservation of a small population through the use of semen of sires less related to the animals in the live population [11]. This might be very effective in keeping inbreeding at a low level. Genomic information, such as marker genotypes or genome sequences, can increase the usefulness of cryo-collections [12]: it allows for a (1) more accurate characterisation of potential individuals for cryo-conservation and selection of core collections that contain more diversity than if based solely on pedigree information; (2) more detailed characterisation of cryo-collections, both in terms of diversity stored as well as specific alleles and haplotypes conserved; and (3) more accurate selection of cryo-conserved material to use for introgression of specific alleles and haplotypes or to reconstruct a breed.

In this Special Issue, you will find the results of recent studies that illustrates that the application of genomic tools in improves the accuracy of decisions to be made in the conservation of animal genetic diversity.
